# Peer Review of “Evaluating Population Density as a Parameter for Optimizing COVID-19 Testing: Statistical Analysis”

**DOI:** 10.2196/27257

**Published:** 2021-02-03

**Authors:** Ayman A Allam

**Affiliations:** 1 Faculty of Medicine Zagazig University Zagazig Egypt

**Keywords:** infectious diseases, COVID-19, SARS-CoV2, coronarvirus

*This is a peer review submitted for the paper “Evaluating Population Density as a Parameter for Optimizing COVID-19 Testing: Statistical Analysis.”*

## Round 1 Review

### General Comments

In this paper [1], the authors prospectively analyzed COVID-19 data obtained from 67 Alabama counties using testing realignment along population density instead of density agnostic per capita. They concluded that adjusting the distribution of testing capacity to also account for population density will improve monitoring and response to blunt the speed and spread of the virus.

Generally, the manuscript is properly structured and well understood.

### Specific Comments

#### Minor Comments

1. Change the subtitle “Policy Proposal” to “Introduction” or “Background.”
